# The "Pyroxyline" Base for Artificial Dentures

**Published:** 1871-03

**Authors:** 


### SELECTED ARTICLES.

### ARTICLE IV.

### *The "Pyroxyline" Base for Artificial Dentures.*

The material is cotton fibre, decomposed with ether. It it is not explosive, but combustible at about three hundred degrees Fahrenheit. Hence its name, Pyroxyline. It has the strength, texture, and appearance of translucent horn ; but is more compressible. It is not at all affected by the secretions of the mouth. Raised or pressed between dies at a sissing heat, under a severe pressure, it readily takes and retains the form of the dies. Parts or pieces are readily united by applying to surfaces in contact, ether of the proper strength. If the ether be pure (as is Squibs'), one-third alcohol must be used. All other is uncertain, and is to be tested before trusted. To unite the teeth, filings from the plate, or other like material, are used as a solder; and in all cases the eye must witness an actual solution; the grains or filings must be lost, and a homogeneous lustrous appearance taken on.

But this material, being wholly or partially dissolved, shrinks more than a hundred per cent. in drying. Hence its manipulation throughout, must be treated with reference to this fact. Properly manipulated, its shrinkage is made useful instead of hurtful. The plate never tarnishes, nor will the filings, if the following rules be well observed:

*First.* The filings must be clean, fine, and free from all admixture, except a very little vermillion.

*Second.* They must be thoroughly dissolved, so as to assume a clear translucent and waxy appearance.

*Third.* They must be very thoroughly packed or pressed together, while setting or hardening, to prevent porosity.

*Fourth.* The piece must never be put in the mouth, or in water, until the ether is entirely dried out by a heat equal to boiling water, and continued for at least half an hour after the case is otherwise ready for the mouth. With these precautions, the secretions of the mouth produce no change, except a very slight but agreeable change in the color, leavin a beautiful golden hue, and with a clear and high polish. The pyroxyline acquires additional solidity and strength for six months.

The plates being supplied, well seasoned, they have only to be raised with dies,as are metallic plates, but always with a screw power, never by blows. The male die should be zinc, its counterpart lead.

The impression being taken and the teeth selected, the first thing is to back them up, by putting a quantity of fillings on and about the pins; then dissolve thoroughly with ether; let them dry for half an hour or more; see that the heads of the pins are uncovered, that the material may shrink around the necks of the pins. Use only teeth with good necks and heads.

*Second.* The teeth being thus packed up, may be ground and articulated. When articulated and set to wax, as upon metallic plates, then remove all wax from the outside, wash the plate with alcohol; scratch, serrate, or furrow the surface wherever the filings are to attach. Then put on a thin coat of filings *outside* the teeth; dissolve them well with ether. After a few minutes add more filings, and dissolve them. After filling up to your satisfaction upon the outside of the teeth, put up the case in plaster just as for soldering, only be more careful to carry the plaster over the cutting surfaces of the teeth, to hold them very fast in all directions; and let the plaster cover the inside of the plate quite up to where your filings are to cover it, to prevent the springing of the plate. Always put up your teeth on a true cast of the mouth, and keep them cramped or well fastened to it till the case is put in the mouth.

*Third.* You will now remove the wax, clean and serrate, or furrow the plate; then put on a thin coat of filings under the teeth and over the plate as far as you wish them to extend when finished. Wet plentifully and constantly with ether until they are thoroughly dissolved and well united to the plate. Be careful in putting on the first filings, to press them before wetting, and also after partially drying, very thoroughly down to or under the blocks or teeth, and in each succeeding application repeat with force this pressure. Be sure to make all solid under and around the teeth. The first coat being dry, add another, and yet another, until you have filled up to your pleasure, allowing each coat to dry by itself, and subjecting each to hard pressure. Be careful to keep a space between the plate and teeth open until the finishing coat, and also, do not unite the blocks or teeth until the finishing coat is put on, so that the material may shrink toward the centre and around the pins of each block, and also shrink to the plate without drawing in the teeth. Continue hard pressure as long as you can produce any effect; be sure and make all solid.

All done as above, remove the plaster. This may be done by cutting down to the teeth in the centre of the arch. Each side will thus pry off, leaving the teeth on the original cast. Now fill up the outside, and add another coat of clean, fine, properly colored filings; attend to all imperfections; burnish with a heavy hand all the filings; brush and finish up with the pumice and whitening. Then cramp or screw your case fast to the original cast, to remain until called for. The plate can be fitted to the mouth just as a metallic plate. It will be seen in every step of the process of manipulations, reference is had to the shrinking tendencies of the material:

*First* Hence, the plates are got up and submitted to a ten tons pressure, and left to season for weeks before they are put into the market.

*Second.* When raised or put up by the dentist, they should be pressed, and for a time remain under the pressure of heated dies.

*Third.* When finished and ready for the mouth, they should be submitted to a heat, equal to boiling water, for at least a half hour, to dissipate all remains of ether and shrinkage.

*Fourth.* The filings are first put upon the teeth and pins in order to dry and shrink to the pins and not from them.

*Fifth.* The filings we first put upon the *outside*, that they may shrink up to and around the teeth, and that the inside may reach to and shrink together.

*Sixth.* The filings are to be hard pushed and parted inside, so as to unite them with the outside, and make all solid.

*Seventh.* The filings are to be kept cut off from the base inside, and also the blocks kept separate, until well dried, so as to prevent drawing the teeth out of place..

*Eighth.* A piece should never be put in the mouth, until after being thoroughly dried and free of all taste and smell of ether. Thus there will be no change in the mouth.

For under sets, it is necesary to have the plate in a large sheet, to be cut to the case. And to prevent the danger of springing or drawing together, a very narrow plate of silver should be raised and put over the pyroxyline plate, under the teeth, not so wide but the filings will entirely cover it and hold it fast. This gives the most perfect under set ever made, all our previous views of weight notwithstanding.

A strict adherence to the above instructions will prevent all difficulties of manipulation.—*Canada Journal of Dental Science.*

---

